# Social and Behavioral Risk Marker Clustering Associated with Biological Risk Factors for Coronary Heart Disease: NHANES 2001–2004

**DOI:** 10.1155/2014/389853

**Published:** 2014-02-25

**Authors:** Nicholas J. Everage, Crystal D. Linkletter, Annie Gjelsvik, Stephen T. McGarvey, Eric B. Loucks

**Affiliations:** ^1^Department of Epidemiology, Brown University School of Public Health, Providence, RI 02912, USA; ^2^Center for Statistical Sciences, Brown University School of Public Health, Providence, RI 02912, USA; ^3^Department of Biostatistics, Brown University School of Public Health, Providence, RI 02912, USA; ^4^Diabetes Prevention and Control Program, Rhode Island Department of Health, Providence, RI 02912, USA; ^5^International Health Institute, Brown University School of Public Health, Providence, RI 02912, USA; ^6^Center for Population Health & Clinical Epidemiology, Brown University School of Public Health, Providence, RI 02912, USA

## Abstract

*Background*. Social and behavioral risk markers (e.g., physical activity, diet, smoking, and socioeconomic position) cluster; however, little is known whether clustering is associated with coronary heart disease (CHD) risk. Objectives were to determine if sociobehavioral clustering is associated with biological CHD risk factors (total cholesterol, HDL cholesterol, systolic blood pressure, body mass index, waist circumference, and diabetes) and whether associations are independent of individual clustering components. *Methods*. Participants included 4,305 males and 4,673 females aged ≥20 years from NHANES 2001–2004. Sociobehavioral Risk Marker Index (SRI) included a summary score of physical activity, fruit/vegetable consumption, smoking, and educational attainment. Regression analyses evaluated associations of SRI with aforementioned biological CHD risk factors. Receiver operator curve analyses assessed independent predictive ability of SRI. *Results*. Healthful clustering (SRI = 0) was associated with improved biological CHD risk factor levels in 5 of 6 risk factors in females and 2 of 6 risk factors in males. Adding SRI to models containing age, race, and individual SRI components did not improve C-statistics. *Conclusions*. Findings suggest that healthful sociobehavioral risk marker clustering is associated with favorable CHD risk factor levels, particularly in females. These findings should inform social ecological interventions that consider health impacts of addressing social and behavioral risk factors.

## 1. Introduction

Coronary heart disease (CHD) is the leading cause of death in the United States, despite extensive gains in primary and secondary prevention [1, 2]. Social factors, including education, may be important risk markers for CHD [3–6]. Better educated and wealthier groups have achieved significant decreases in CHD risk factors compared to their less educated, poorer counterparts [7]. Furthermore, there has been emphasis on social ecological intervention models, that take into account the social context such as socioeconomic position (e.g., education), race/ethnicity, neighborhood characteristics and social network transmission of health behaviors, which may shape the success of health behavior interventions or the behaviors themselves [8–12]. Also, lifestyle risk factors, such as smoking, low physical activity, low fruit/vegetable intake, low fiber intake, and high trans-fat and saturated fat diets have evidence to be responsible for a substantial proportion of CHD events [13–19]. While a number of individual- and community-based trials, including the Stanford Five-City Project, the Pawtucket Heart Health Program, and the Multiple Risk Factor Intervention Trial (MRFIT), have attempted to change lifestyle risk factors/behaviors related to CHD [20–25], systematic reviews on cardiovascular disease prevention interventions have shown little to modest favorable reductions in cardiovascular disease risk in response to these programs [25, 26]. One method for improving interventions may be focusing on multiple social and behavioral risk factors at a time. Previous work has shown that social and behavioral risk markers (including physical activity, diet, smoking and educational attainment) cluster [27], but it is not known whether the clustering is related to CHD risk or whether any associations with CHD risk are independent of the individual social and behavioral risk marker components. In considering interventions to prevent CHD, it may be helpful to consider the potential mutually reinforcing characteristics of both social and behavioral risk markers. This could facilitate the creation of more effective interventions, for example, if interventions on a single risk marker (e.g., physical activity) were substantially affected by other co-occurring risk factors such as diet, smoking and socioeconomic position. Understanding which social and behavioral risk factors might mutually influence each other could substantially inform etiologic understanding of CHD, and identify possible interventions aimed at addressing the mutually reinforcing causes of CHD. Comparable research on the metabolic syndrome, another clustering of conditions relevant to CHD and diabetes, has demonstrated that while the biological CHD risk factors (e.g., blood pressure, central obesity, fasting glucose and lipids) do co-occur more often than would be expected due to chance (i.e., cluster), the clustering does not appear to confer risk above its individual components [28–33]. Therefore, the objective of this study was to evaluate whether sociobehavioral clustering is associated with biological CHD risk factors including hypertension, dyslipidemia, obesity, and diabetes in the National Health and Nutrition Examination Survey (NHANES) 2001–2004. In addition, we aimed to determine if the clustering itself is associated with biological CHD risk factors independently of the individual contributions of each social and behavioral risk marker.

## 2. Methods

### 2.1. Study Sample

The study included participants from the 2001-2002 and 2003-2004 NHANES. Participants were interviewed in their homes and in mobile examination centers across the United States. The study sample for this analysis included all participants ≥20 years old (*N* = 10,452). Participants missing data on education (*n* = 22), smoking status (*n* = 35), physical activity (*n* = 306), and/or fruit and vegetable intake (*n* = 1,260) were excluded from analyses. The high number of missing fruit and vegetable intake data was primarily due to participants either not completing dietary recalls due to refusal, having incomplete information on diet, or arriving late to the NHANES mobile examination centers with resulting insufficient time to complete dietary assessments. The final analytic sample size was 8,798. All participants had at least one criteria for determining diabetes disease status. However, there were missing data on body mass index (*n* = 261), total cholesterol (*n* = 444), high-density lipoprotein cholesterol (HDL) (*n* = 445), and systolic blood pressure (*n* = 371). The dependent variable with the median amount of missing data was systolic blood pressure. Therefore, we included this variable in our comparison analysis of included and excluded participants. Compared to excluded participants, included participants were younger (49 versus 56 years; *P* < 0.0001), had slightly higher smoking prevalence (22.6% versus 19.5% smokers; *P* = 0.004), were less likely to have attended college (53.1% versus 61.7% with > high school education; *P* < 0.0001), and were more likely to meet physical activity guidelines (52.9% versus 44.0% met guidelines; *P* < 0.0001). Included participants were more likely to be non-Hispanic white (53.3% versus 52.1%) or Mexican American/other Hispanic race/ethnicity, and less likely to be non-Hispanic black (18.9% versus 20.8%) or “other” race/ethnicity (3.7% versus 5.3%; *P* = 0.0007). Included participants had slightly lower systolic blood pressure (125.9 versus 130.8 mmHg; *P* < 0.0001), waist circumference (97.3 cm versus 98.7 cm; *P* = 0.03), HDL (53.4 versus 54.5 mg/dL; *P* = 0.05), less obesity (31.5% versus 34.6%; *P* = 0.05) and less likely to have diabetes (11.6% versus 14.2%; *P* = 0.002) than excluded participants; however there were no differences in total cholesterol (203.1 versus 201.2 mg/dL; *P* = 0.20), or fruit and vegetable guideline compliance (53.7% versus 56.1% met guidelines; *P* = 0.26).

### 2.2. Biological Coronary Heart Disease (CHD) Risk Factors

#### 2.2.1. Lipids

Total cholesterol was determined enzymatically using the Trinder-type method [34]. HDL cholesterol was assessed using standard enzymatic assays; molecules were separated from plasma by chemical precipitation with dextran sulfate-magnesium, and the resulting supernatant was assayed for cholesterol [35, 36]. The NHANES quality control and quality assurance protocols met the 1988 Clinical Laboratory Improvement Act mandates. Participants were classified as having dyslipidemia according to National Cholesterol Education Program (NCEP) defined cut-points for HDL levels of <40 mg/dL in males, <50 mg/dL in females, and total cholesterol levels ≥240 mg/dL in males and females [37].

#### 2.2.2. Systolic Blood Pressure

Certified physician examiners conducted three blood pressure measurements after having participants rest in a seated position for 5 minutes [38]. The training of physician examiners as well as extensive documentation on the quality control measures are found in the NHANES physical examination protocol [38]. The mean of the second and third systolic blood pressure measurements was used for analyses. Participants with systolic blood pressure ≥140 mmHg were classified as having hypertension according to the 2003 Seventh Report of the Joint National Committee on Prevention, Detection, Evaluation, and Treatment of High Blood Pressure [39].

#### 2.2.3. Obesity

Obesity was determined by body mass index (BMI) and waist circumference. Body weight was measured to the nearest 0.1 lb with the use of a daily calibrated Toledo digital scale and with subjects wearing only disposable paper gowns and slippers [40]. This measurement was then converted to kilograms. Height was measured with the use of a stadiometer (to the nearest 0.25 in, and then converted to meters) [40]. BMI was calculated as the weight in kilograms divided by the square of the height in meters (kg/m^2^). Participants with a BMI ≥30 kg/m^2^ were classified as obese. With participants standing and having normally expelled air, trained examiners measured participants' waist circumference using a tape measure placed at the lateral border of the ilium [40]. Participants who exceeded waist circumference guidelines (males: >94 cm; females: >80 cm) were considered to have large waist circumference [41].

#### 2.2.4. Diabetes

Presence of diabetes was defined as self-reported, doctor-diagnosed diabetes; self-reported use of oral glucose-lowering medications; self-reported insulin use; visually confirmed pharmaceutical use of oral glucose-lowering drug and insulin; or fasting plasma glucose ≥126 mg/dL. Plasma glucose was collected from participants after a 12-hour fast before the examination. Glucose levels were measured using the hexokinase ultraviolet method [42]. The NHANES quality control and quality assurance protocols met the 1988 Clinical Laboratory Improvement Act mandates.

### 2.3. Social and Behavioral Risk Marker Clustering Variables

The social independent variable (education) and the three behavioral independent variables (diet, physical activity, smoking) were selected *a priori.* Physical activity, diet and smoking were selected due to evidence that they are three of the most important behavioral risk factors for CHD [15–17]. With regard to social factors, socioeconomic position was selected as it has been demonstrated to be consistently associated with CHD in systematic reviews [18]. Of the different measures of SEP (e.g., income, occupation, education), we selected education as it remains stable across adulthood, participants are more likely to agree to report on it compared to income for which there are more missing data, and it is a variable that can influence other SEP measures such as income and occupation [19].

#### 2.3.1. Physical Activity

The relevant national physical activity guidelines for the period from 2001–2004 were those by the Centers for Disease Control and the American College of Sports Medicine [43]. The guidelines stated that “adults should accumulate 30 minutes or more of moderate-intensity physical activity on most, preferably all, days of the week” [43]. Due to a lack of specificity in the guidelines language, we interpreted the recommendations as suggesting that adults should exercise at least 5 days per week for at least 30 minutes, or ≥150 minutes of moderate and/or vigorous of physical activity per week [44]. Estimates of weekly physical activity were based on participant responses to the following questions: “Over the past 30 days, have you walked or bicycled as part of getting to and from work, or school, or to do errands?” and “Over the past 30 days, did you do any tasks in or around your home or yard for at least 10 minutes that required moderate or greater physical effort [45]?” Participants also reported time and effort spent on 45 leisure-time activities, such as gardening, weightlifting, walking, swimming, and yoga [46]. The average duration and frequency for each activity was reported and used to estimate the weekly physical activity time for each participant.

#### 2.3.2. Fruit and Vegetable Intake

Fruit and vegetable consumption in NHANES was measured using 24-hour dietary recalls [47, 48]. The recalls follow methodology based on the joint-venture program “What We Eat in America” established by the U.S. Department of Agriculture (USDA) and the Department of Health and Human Services [49]. From 2001-2002, NHANES collected one 24-hour recall, but in 2003-2004 expanded collection to one 24-hour recall in the Mobile Examination Center and an additional recall 3–10 days later. In 2003-2004, approximately 87% of the sample completed both dietary recalls. Based on the dietary recall, the USDA calculated the number of cups of each food group consumed by the participant using the MyPyramid Equivalents Database (MPEDS). The 1992 Food Guide Pyramid recommendations were still in effect during the 2001–2004 time period of the analysis. Therefore, we considered the participants as having met the guidelines if they consumed ≥3 servings (1.5 cups) of vegetables and ≥2 servings (1 cup) of fruit per day [50].

#### 2.3.3. Smoking Status

Participants reporting smoking <100 cigarettes in their life or no longer smoking were considered nonsmokers. We classified participants who reported smoking “every day” or “some days” as current smokers.

#### 2.3.4. Socioeconomic Position

Participants reported their highest level of educational attainment and, based on previous literature [51, 52], we operationalized the variable as ≤ high school (i.e., high school diploma, General Equivalency Diploma (GED), or less) versus > high school (e.g., some college, associate's degree, college or postgraduate).

### 2.4. Covariates

Participants reported their age, sex, and race/ethnicity during the home visit of NHANES. The categories for race/ethnicity were: non-Hispanic black (*n* = 1,719), non-Hispanic white (*n* = 4,752), Mexican-American/other Hispanic (*n* = 2,174), or “other race” (*n* = 333). Patients also reported if they were currently taking antihypertensive medications and/or cholesterol-lowering medication.

### 2.5. Statistical Analyses

Each of the sociobehavioral clustering variables (i.e., smoking status, meeting fruit and vegetable and physical activity guidelines, and education attainment) were dichotomized into indicator variables (i.e., 1 = Yes, 0 = No) using cut points described above. A Sociobehavioral Risk Marker Index (SRI) ranging from 0–4 (0 = No risk factors; 4 = All risk factors) was constructed by summing the indicator variables. Statistically significant clustering has been identified elsewhere using the SRI [27].

Multivariable-adjusted linear and logistic regression analyses compared SRI = 0 and SRI = 4 to having 1, 2 or 3 risk markers (SRI = 1–3), in order to evaluate associations of healthful (SRI = 0) and unhealthful (SRI = 4) risk marker clustering with biological CHD risk factors. Similar analyses have been conducted to assess the risk conferred by the metabolic syndrome over its individual components with incident CHD outcomes (e.g., CHD-related mortality) [30, 31]. Because the NHANES data are cross-sectional and lack incident CHD information, analyses used C-statistics from receiver operating curves (ROC) to evaluate the predictive ability (i.e., comparison of the sensitivity and false positive proportions) of the of the SRI on the CHD biological risk factors [30, 31]. Sex-, racial/ethnic- and age-stratified analyses were used to understand potential effect modification of the associations between the SRI and the CHD biological risk factors. Formal statistical testing for interactions demonstrated significant interactions between the SRI = 0 (versus 1, 2, 3) and sex for 3 of the 6 CHD biological risk factor outcomes, specifically HDL (*P* = 0.002), waist circumference (*P* < 0.0001) and obesity (*P* < 0.0001). Statistically significant interactions were also found between SRI = 4 (versus 1, 2, 3) and sex for HDL (*P* = 0.003) and waist circumference (*P* = 0.04). Consequently analyses were performed sex-specific. With regard to race/ethnicity, only one of twelve statistical tests (interaction with HDL (*P* = 0.03); for the 6 biological CHD risk factors in males and females separately) for interactions between SRI and race/ethnicity were significant (*P* < 0.05), consequently race/ethnicity-specific analyses were not performed. All analyses were adjusted for race/ethnicity. Statistical analyses were conducted using SAS 9.2 (Carey, NC).

## 3. Results

**Figure 1:**
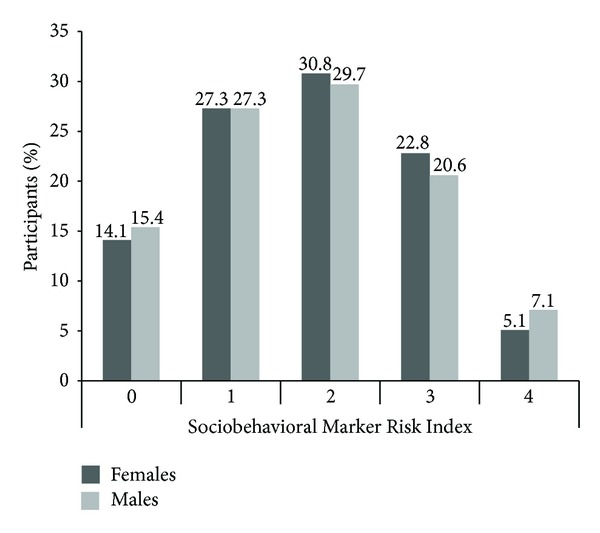
Distribution of Sociobehavioral Risk Marker Index (SRI) by Sex, NHANES 2001–2004.

Characteristics of the study sample are shown in Table 1 and the distribution of the SRI by sex is described in Figure 1. Females demonstrated higher BMI, total cholesterol, and HDL cholesterol, but lower smoking, systolic blood pressure, waist circumference, cholesterol medication use, physical activity guideline compliance and fruit/vegetable consumption guideline compliance than males. There were no significant differences between sexes for diabetes, antihypertensive medication use, education, race/ethnicity or age.

The individual components of the SRI (i.e., education, smoking, fruit and vegetable consumption and physical activity) were associated with the biological CHD risk factors fairly extensively in females and less so in males (Table 2). Specifically, of the 6 biological CHD risk factors that were evaluated (i.e., systolic blood pressure, total cholesterol, HDL cholesterol, waist circumference, body mass index and diabetes), the SRI components education, smoking, fruit/vegetable consumption and physical activity were associated with 5, 1, 3 and 4 of the biological CHD risk factors, respectively, in females, and were associated with only 2, 2, 0 and 3 of the biological CHD risk factors, respectively, in males (Table 2).

Healthful clustering of SRI components was strongly associated with more favorable biological CHD risk factors in females (5 of 6 CHD risk factors were associated with SRI = 0 versus SRI = 1–3) and less so in males (2 of 6 risk factors were associated with SRI = 0 versus SRI = 1–3; Table 3). In an effort to evaluate if the clustering itself was associated with the biological CHD risk factors over and above the individual contributions of SRI components, analyses additionally adjusted for the individual SRI components, and generally showed substantial reductions in effect size, suggesting that the clustering itself was not overarchingly associated with CHD risk factors independently of the individual SRI components (Table 3). Unhealthy clustering of SRI components (i.e., SRI = 4) was not substantially associated with CHD risk factors in females or males (Table 3).

ROC curve analyses utilizing C-statistics demonstrated predictive ability for SRI in relation to all biological CHD risk factors, although the SRI predictive ability was particularly high for systolic hypertension and diabetes (Table 4). In an effort to evaluate if the clustering associated with SRI = 4 or SRI = 0 contributed to biological CHD risk factors levels over and above the individual SRI components themselves (i.e., education, smoking, fruit/vegetable consumption and physical activity), three different C-statistics were calculated that included age, race/ethnicity and (1) individual SRI components only, (2) SRI only, or (3) both SRI and individual SRI components. As shown in Table 4, generally there was very minimal change in C-statistic values in models that included both SRI and the individual SRI components, compared with models that included only the SRI, or the individual SRI components only (in addition to age and race/ethnicity). These findings suggested that the SRI clustering itself did not contribute to biological CHD risk factor levels over and above age, race/ethnicity and the individual SRI components. Similarly, the individual SRI components did not contribute to biological CHD risk factor levels independently of age, race/ethnicity and the SRI clustering values of SRI = 0 or SRI = 4, versus SRI = 1–3.

## 4. Discussion

Overall findings demonstrated that healthful SRI clustering was associated with improved biological CHD risk factor (systolic blood pressure, HDL cholesterol, waist circumference, BMI and diabetes) levels in females, and less so in males (healthful SRI clustering associated only with HDL cholesterol and diabetes). Unhealthy SRI clustering generally was not associated with biological CHD risk factors. Furthermore, findings suggested that the SRI clustering does not affect biological CHD risk factors independent of its individual components.

### 4.1. Prior Research

To our knowledge, no other studies have evaluated associations of social and behavioral risk marker clustering with biological CHD risk factors. However, other measures of biological CHD risk factor clustering, specifically the metabolic syndrome, have been well demonstrated to be related to both incident CHD and diabetes [30–32]. However, similarly to the sociobehavioral clustering described here, the metabolic syndrome does not appear to predict CHD risk independently of its individual components [30, 31, 53, 54].

### 4.2. Potential Mechanisms

The SRI components (i.e., education, smoking, fruit and vegetable consumption and physical activity) were generally related to CHD biological risk factors. Specifically, education was generally inversely associated with systolic blood pressure [5, 7, 55–58], waist circumference [59], body mass index [55, 56] and diabetes [7, 60], positively associated with HDL cholesterol [61, 62], and not related to total cholesterol [7, 62]. Furthermore, smoking was overall positively associated with systolic blood pressure [63], total cholesterol [64, 65], waist circumference [66], and diabetes [67, 68], and inversely associated with HDL [65]. It should be noted that those who smoke heavily are generally more likely to be obese, but light and moderate smokers have decreased risk of obesity [66]. Studies have demonstrated that fruit and vegetable intake is typically inversely associated with systolic blood pressure in men and women [69, 70] and waist circumference in women [71], but evidence suggests a lack of association of fruit and vegetable consumption with total cholesterol [72] and diabetes [73–75]. Research is also equivocal on fruit and vegetable intake's effect on HDL and obesity [71, 76–79]. The weakness or lack of association between fruit and vegetable intake and blood lipid levels may reflect the need to account for other aspects of diet, including healthful oil and fiber consumption [77, 80–82]. Finally, physical activity has been inversely associated with systolic blood pressure [83, 84], waist circumference [85, 86], obesity [87–89], and diabetes [90, 91]; and positively associated with HDL [82, 92]. Evidence for an inverse effect between physical activity and total cholesterol is not consistent [82, 93].

Gender differences were observed, where in females the SRI was associated with systolic blood pressure, HDL cholesterol, waist circumference, BMI and diabetes, while in males the SRI was associated with HDL cholesterol and diabetes. Similar gender differences have been seen in the education-health literature where associations of education with CHD, diabetes and metabolic syndrome are often stronger in females than males [18, 94–97]. Potential mechanisms for gender differences include obesity-related effects on social mobility and greater concurrent psychosocial risk for low socioeconomic position women than men. For example, obesity was reported to be stigmatized more highly in women than men, and obesity may limit upward social mobility more so in women than men [98]. Furthermore, findings demonstrate that women with low education level have additional psychosocial risks (including high depressive symptoms, income under the poverty threshold, unemployment, and single parenting) than men with low education [56]. This greater psychosocial burden in low socioeconomic position women may be associated in turn with poorer metabolic outcomes, as suggested in other studies that showed a relation between depression and metabolic syndrome [99, 100] and income and metabolic syndrome [101, 102]. Future research focused on identifying mechanisms responsible for gender differences in the relation between sociobehavioral clustering and CHD risk will provide better knowledge of the potential pathways.

There have been substantial advancements emphasizing the importance of social ecological intervention models that take into account the social context which may shape the success of health behavior interventions, or the behaviors themselves [8–10, 12, 103]. In considering interventions to prevent CHD, it may be helpful to consider the mutually reinforcing characteristics of both social and behavioral risk factors. This could help to create more effective interventions. For example, if interventions on a single risk factor (e.g., physical activity) may be substantially affected by co-occurring other risk factors such as diet, smoking and socioeconomic position. Furthermore, as social and behavioral risk marker clustering has been demonstrated to occur [27], it is also important to determine whether that clustering confers risk for CHD. This study demonstrated that healthful sociobehavioral clustering was associated with biological CHD risk factors particularly in females, however the clustering itself did not confer risk over and above the individual social and behavioral risk markers themselves. Despite a lack of additional risk beyond its individual components, the SRI may still provide value to clinicians and public health researchers. Specifically, sociobehavioral clustering occurs, which may be important when devising interventions to prevent or treat the effects of the individual SRI components (education, fruit and vegetable intake, physical activity and smoking). Furthermore, healthful sociobehavioral clustering is related to improved biological CHD risk factor levels, particularly in females. This improved biological CHD risk factor levels are likely due to the individual contributions of education, physical activity, fruit and vegetable consumption and lack of smoking.

### 4.3. Limitations

This study assessed the risk conferred by the SRI using biological CHD risk factors, which are estimates of future risk for CHD. Future studies should evaluate associations of the SRI with clinical endpoints such as incident myocardial infarction, type 2 diabetes or mortality to better understand the importance of sociobehavioral CHD risk factor clustering with health outcomes [30, 31]. Furthermore, our study used cross-sectional data, which limits causal inference for the relation between sociobehavioral clustering and biological CHD risk factors. Additionally, recall of physical activity and fruit and vegetables intake has substantial measurement error, suggesting that a reasonable amount of misclassification occurred [104]. Multiple 24-hour recalls are preferred over using dietary data from a single 24-hour period. In our study, NHANES increased the number of dietary recall days from 1 day in 2001-2002 to 2 days in 2003-2004. A strength of this study was the use of NHANES data, with its high level of quality control and quality assurance [105].

## 5. Conclusion

This study suggests that, particularly among women, healthful sociobehavioral risk marker clustering is related to having more favorable levels of CHD risk factors. Sociobehavioral clustering itself was not related to CHD risk independent of the individual clustering components, suggesting it may be the individual sociobehavioral risk factors themselves (i.e., physical activity, diet, smoking and education) that are responsible for associations with CHD risk. As social ecological intervention models advance, and take into account the social context which may shape the success of health behavior interventions [8–10, 12, 103], it will be important to carefully consider the mutually reinforcing characteristics of both social and behavioral risk factors. This should help to create more effective interventions. As social and behavioral risk marker clustering has recently been demonstrated to occur [27], it is also important to determine whether that clustering confers risk for CHD. The current study's findings should further inform social ecological interventions that consider the potential health impacts of addressing both social and behavioral risk factors.

## Figures and Tables

**Table 1:** Descriptive characteristics of study participants stratified by sex, NHANES 2001–2004.

	Men (*n* = 4,305)		Women (*n* = 4,673)	
	Mean or proportion	95% CI*	Mean or proportion	95% CI
Age				
20–29 years, %	17.7	15.0–20.4	20.5	18.0–23.1
30–39 years, %	15.7	13.0–18.4	18.4	15.8–21.0
40–49 years, %	18.0	15.3–20.7	16.4	13.8–19.0
50–59 years, %	13.7	10.9–16.5	12.1	9.4–14.8
60–69 years, %	14.7	12.0–17.5	14.7	12.0–17.3
70–79 years, %	12.7	9.9–15.5	9.9	7.1–12.6
≥80 years, %	7.4	4.6–10.3	8.0	5.3–10.8
Race/ethnicity				
Non-Hispanic white, %	53.0	50.9–55.0	52.9	50.9–54.9
Non-Hispanic black, %	19.2	16.5–21.9	19.1	16.5–21.7
Mexican-American/other Hispanic, %	24.4	21.8–27.0	24.0	21.5–26.5
Other race, %	3.4	0.5–6.4	4.0	1.2–6.8
Education				
≤High school diploma/GED, %	54.2	52.1–56.2	52.5	50.5–54.5
>High school diploma/GED, %	45.8	43.6–48.0	47.5	45.4–49.6
Current smoker, %	27.2	24.6–29.7	18.4	15.8–21.0
Did not meet fruit and vegetable guidelines, %^†^	51.7	49.6–53.8	55.4	53.5–57.3
Did not meet physical activity guidelines, %^‡^	43.8	41.5–46.0	51.2	49.2–53.2
Current diabetes, %**	12.7	9.9–15.5	10.9	8.2–13.6
Systolic blood pressure, mmHg	126.7	126.2–127.3	125.0	124.4–125.7
Taking antihypertensive medication, %	22.4	19.8–25.1	23.6	21.1–26.1
Total cholesterol, mg/dL	200.0	198.5–201.3	206.1	204.8–207.4
HDL cholesterol, mg/dL	47.5	47.1–47.9	58.9	58.4–59.4
Taking cholesterol-lowering medication, %	20.5	17.2–23.7	16.4	13.2–19.5
Body mass index (kg/m^2^)	27.9	27.7–28.1	28.7	28.5–28.9
Waist circumference (cm)	99.7	99.3–100.1	95.5	95.0–95.9

*Confidence interval.

^†^Food Guide Pyramid guidelines recommended consuming at least 3 servings of vegetables and 2 servings of fruits each day.

^‡^Centers for Disease Control/ American College of Sports Medicine guidelines recommended moderate exercising at least 30 minutes most days of the week.

**Defined as self-reported, doctor-diagnosed diabetes; self-reported use of oral glucose-lowering medications; self-reported insulin use; visually confirmed pharmaceutical use of oral glucose-lowering drug and insulin; or fasting plasma glucose ≥126 mg/dL.

**Table 2:** Associations of individual components of the Sociobehavioral Risk Marker Index (i.e., education, smoking, fruit/vegetable consumption, and physical activity) with biological coronary heart disease risk factors, stratified by sex and adjusted for race/ethnicity and age, NHANES 2001–2004.

	Coronary heart disease risk factors											
	Average systolic blood pressure (mmHg)*		Total cholesterol (mg/dL)^†^		HDL cholesterol (mg/dL)^†^		Waist circumference (cm)		Obese (yes/no)^‡^		Diabetes (yes/no)^§^	
	β**	95% CI^††^	*β*	95% CI	*β*	95% CI	*β*	95% CI	OR^‡‡^	95% CI	OR	95% CI
Females (*n* = 4,673)												
≤High school versus >high school	**1.22**	**0.13, 2.32**	0.84	−2.24, 3.92	−**3.58**	−**4.83, **−**2.33**	**3.25**	**2.33, 4.17**	**1.34**	**1.18, 1.52**	**1.49**	**1.21, 1.83**
Smoker versus nonsmoker	0.61	−0.78, 2.00	0.77	−3.31, 4.86	−**5.40**	−**7.06, **−**3.75**	−0.37	−1.55, 0.80	0.99	0.84, 1.16	1.25	0.95, 1.64
Did not meet versus met fruit and vegetable guidelines	**2.48**	**1.42, 3.55**	−1.11	−4.04, 1.82	−**2.50**	−**3.70, **−**1.31**	0.35	−0.55, 1.25	**1.14**	**1.01, 1.29**	1.02	0.84, 1.23
Did not meet versus met physical activity guidelines	0.41	−0.67, 1.49	1.50	−1.46, 4.47	−**3.28**	−**4.89, **−**2.08**	**3.95**	**3.05, 4.85**	**1.41**	**1.24, 1.59**	**1.29**	**1.06, 1.58**
Males (*n* = 4,305)												
≤High school versus >high school	**1.73**	**0.71, 2.75**	−1.08	−4.66, 2.50	−**1.40**	−**2.40, **−**0.41**	−0.58	−1.45, 0.29	0.94	0.82, 1.08	1.04	0.86, 1.27
Smoker versus nonsmoker	0.92	−0.22, 2.05	1.62	−2.75, 5.99	0.30	−0.91, 1.52	−**2.49**	−**3.45, **−**1.53**	**0.71**	**0.61, 0.84**	1.06	0.84, 1.33
Did not meet versus met fruit and vegetable guidelines	0.94	−0.05, 1.93	1.17	−2.31, 4.66	−0.63	−1.59, 0.34	−0.28	−1.12, 0.56	0.97	0.85, 1.11	1.06	0.88, 1.28
Did not meet versus met physical activity guidelines	0.23	−0.78, 1.24	−1.89	−5.48, 1.70	−**1.43**	−**2.43, **−**0.43**	**1.05**	**0.19, 1.91**	1.09	0.95, 1.26	**1.35**	**1.12, 1.63**

*Adjusted for hypertension medication use.

^†^Adjusted for cholesterol medication use.

^‡^Obesity defined as body mass index ≥30 kg/m^2^ versus <30 kg/m^2^.

^§^Defined as self-reported, doctor-diagnosed diabetes; self-reported use of oral glucose-lowering medications; self-reported insulin use; visually confirmed pharmaceutical use of oral glucose-lowering drug and insulin; or fasting plasma glucose ≥126 mg/dL.

**Coefficient for one unit change in the outcome when comparing levels of the social or behavioral risk marker.

^††^95% confidence interval.

^‡‡^Odds ratio.

**Table 3:** Associations of Sociobehavioral Risk Marker Index (SRI) clustering with coronary heart disease risk factors, NHANES 2001–2004.

	Coronary Heart Disease Risk Factors											
	Average systolic blood pressure (mmHg)*		Total cholesterol (mg/dL)^†^		HDL cholesterol (mg/dL)^†^		Waist circumference (cm)		Obese (yes/no)^‡^		Diabetes (yes/no)	
	*β* ^§^	95% CI**	*β*	95% CI	*β*	95% CI	*β*	95% CI	OR^††^	95% CI	OR	95% CI
Females (*n* = 4,673)												
SRI = 0^‡‡^												
Adjusted for age and race	−**1.81**	−**3.34, −0.27**	−0.30	−4.26, 3.66	**5.34**	**3.74, 6.94**	**−4.50**	**−5.78, −3.21**	**0.59**	**0.48, 0.71**	**0.62**	**0.44, 0.87**
Adjusted for age, race, and SRI individual components	0.88	−1.24, 2.99	−1.02	−6.62, 4.59	1.90	−0.35, 4.16	**−2.03**	−**3.79, −0.27**	**0.75**	**0.58, 0.97**	0.80	0.51, 1.23
SRI = 1–3	0.00	—	0.00	—	0.00	—	0.00	—	1.00	—	1.00	—
SRI = 4												
Adjusted for age and race	0.03	−2.41, 2.46	5.25	−2.52, 13.03	−**6.09**	−**9.24, **−**2.95**	0.12	−1.92, 2.16	0.96	0.73, 1.27	1.35	0.89, 2.07
Adjusted for age, race, and SRI individual components	−2.17	−5.20, 0.86	6.00	−3.40, 15.39	−0.35	−4.13, 3.43	−1.44	−3.96, 1.09	0.82	0.58, 1.16	1.07	0.62, 1.85
Males (*n* = 4,305)												
SRI = 0												
Adjusted for age and race	−1.17	−2.55, 0.21	2.06	−2.37, 6.48	**2.64**	**1.41, 3.87**	−0.61	−1.78, 0.56	0.97	0.81, 1.17	**0.65**	**0.48, 0.87**
Adjusted for age, race, and SRI individual components	0.97	−0.99, 2.92	5.11	−1.42, 11.65	2.71	0.90, 4.52	−**1.69**	−**3.35, **−**0.03**	0.87	0.67, 1.14	0.70	0.47, 1.04
SRI = 1–3	0.00	—	0.00	—	0.00	—	0.00	—	1.00	—	1.00	—
SRI = 4												
Adjusted for age and race	0.42	−1.56, 2.40	−2.42	−10.74, 5.90	−0.19	−2.50, 2.11	−**1.80**	−**3.46, **−**0.14**	0.78	0.59, 1.04	0.88	0.60, 1.29
Adjusted for age, race, and SRI individual components	−1.65	−4.17, 0.87	−7.06	−17.33, 3.22	−0.92	−3.76, 1.93	0.28	−1.84, 2.40	1.02	0.71, 1.46	0.78	0.48, 1.27

*Adjusted for hypertension medication use.

^†^Adjusted for cholesterol medication use.

^‡^Obesity defined as body mass index ≥30 kg/m^2^ versus <30 kg/m^2^.

^§^Coefficient for one unit change in the outcome when comparing levels of the Sociobehavioral Risk Index to the referent group.

**95% Confidence Interval.

^††^Odds ratio.

^‡‡^Sociobehavioral Risk Marker Index (SRI) components include variables for the following: ≤high school diploma/GED versus >high school diploma/GED; met versus did not meet physical activity guidelines; met versus did not meet fruit and vegetable guidelines; smoker versus nonsmoker. Each participant received either a 1 if they had the particular social or behavioral risk marker or a 0 if they did not have the risk marker. The sum of these values produced an index with a range of 0–4, with SRI = 4 indicating having all risk markers (i.e., all unhealthy risk markers) and SRI = 0 indicating that the participant had no risk markers (i.e., all healthy risk markers).

**Table 4:** Predictive ability of the Sociobehavioral Risk Marker Index (SRI) for biological coronary heart disease risk factors, stratified by sex. Three different receiver operating characteristic (ROC) curve analyses, resulting in three different C-statistics, were performed that included age and race/ethnicity as well as (1) individual SRI components only (i.e., education, smoking, fruit/vegetable consumption, and physical activity), (2) SRI only, or (3) both SRI and individual SRI components, NHANES 2001–2004.

	Coronary heart disease risk factors											
	Systolic hypertension (yes/no)*		Elevated total cholesterol (yes/no)^†^		Low HDL cholesterol (yes/no)^‡^		Large waist circumference (yes/no)^§^		Obese (yes/no)**		Diabetes (yes/no)	
	C^††^	95% CI^‡‡^	C	95% CI	C	95% CI	C	95% CI	C	95% CI	C	95% CI
Females												
Only includes SRI^§§^ individual components	0.85	0.84, 0.87	0.59	0.57, 0.62	0.62	0.60, 0.65	0.68	0.65, 0.70	0.57	0.55, 0.59	0.75	0.73, 0.77
SRI = 0 or 4^§§^ versus SRI = 1–3												
Includes SRI only	0.85	0.84, 0.86	0.59	0.56, 0.61	0.60	0.58, 0.62	0.66	0.64, 0.68	0.56	0.54, 0.58	0.75	0.73, 0.77
Includes SRI and SRI individual components	0.85	0.84, 0.87	0.59	0.57, 0.62	0.62	0.60, 0.65	0.68	0.65, 0.70	0.57	0.56, 0.59	0.75	0.73, 0.77
Males												
Only includes SRI individual components	0.78	0.76, 0.80	0.57	0.54, 0.60	0.58	0.55, 0.60	0.68	0.66, 0.70	0.55	0.53, 0.57	0.75	0.73, 0.77
SRI = 0 or 4 versus SRI = 1–3												
Includes SRI only	0.78	0.76, 0.80	0.56	0.53, 0.59	0.57	0.54, 0.59	0.68	0.66, 0.69	0.51	0.49, 0.53	0.75	0.73, 0.76
Includes SRI and SRI individual components	0.78	0.76, 0.80	0.57	0.55, 0.60	0.58	0.56, 0.61	0.68	0.66, 0.70	0.55	0.53, 0.57	0.75	0.73, 0.77

*Systolic hypertension is defined as having systolic blood pressure ≥140 mmHg. Model includes antihypertensive medication use.

^†^Elevated total cholesterol is defined as having total cholesterol ≥240 mg/dL. Model includes cholesterol-lowering medication use.

^‡^Low HDL cholesterol is defined as having HDL <50 mg/dL for women and <40 mg/dL for men. Adjusted for cholesterol medication use.

^§^Large waist circumference is defined as having a waist circumference >88 cm for women and >102 cm for men.

**Obesity defined as ≥30 kg/m^2^ versus <30 kg/m^2^.

^††^C-Statistic determined from Mann-Whitney test for ROC curves.

^‡‡^95% confidence interval.

^§§^Sociobehavioral Risk Marker Index (SRI) components include variables for the following: less than high school or high school diploma/GED versus more than high school; met versus did not meet physical activity guidelines; met versus did not meet fruit and vegetable guidelines; current versus never or former smokers. Each participant received either a 1 if they had the particular sociobehavioral risk marker or a 0 if they did not have the risk marker. The sum of these values produced an index with a range of 0–4, with SRI = 4 indicating having all risk markers (i.e., all unhealthy risk markers) and SRI = 0 indicating that the participant had no risk markers (i.e., all healthy risk markers).
